# Multicentric evidence of emotional impairments in hypertensive heart disease

**DOI:** 10.1038/s41598-020-70451-x

**Published:** 2020-08-24

**Authors:** Adrián Yoris, Agustina Legaz, Sofía Abrevaya, Sofía Alarco, Jéssica López Peláez, Ramiro Sánchez, Adolfo M. García, Agustín Ibáñez, Lucas Sedeño

**Affiliations:** 1 Institute of Cognitive and Translational Neuroscience (INCYT), INECO Foundation, Favaloro University, Buenos Aires, Argentina; 2 National Scientific and Technical Research Council (CONICET), Pacheco de Melo 1860, C1126AAB Buenos Aires, Argentina; 3 Universidad de San Andrés, Buenos Aires, Argentina; 4 Faculty of Health, Santiago de Cali University, Cali, Colombia; 5 Metabolic and Arterial Hypertension Unit, Favaloro Foundation Hospital, Buenos Aires, Argentina; 6 Faculty of Education, National University of Cuyo (UNCuyo), Mendoza, Argentina; 7 Departamento de Lingüística y Literatura, Facultad de Humanidades, Universidad de Santiago de Chile, Santiago, Chile; 8 Global Brain Health Institute, University of California, San Francisco, USA; 9 Universidad Autónoma del Caribe, Barranquilla, Colombia; 10 Center for Social and Cognitive Neuroscience (CSCN), School of Psychology, Universidad Adolfo Ibáñez, Santiago, Chile

**Keywords:** Neuroscience, Psychology, Cardiology

## Abstract

The mechanisms underlying emotional alterations constitute a key research target in neuroscience. Emerging evidence indicates that these disruptions can be related to abnormal interoception (i.e., the sensing of visceral feelings), as observed in patients with cardiodynamic deficits. To directly assess these links, we performed the first multicenter study on emotion recognition and interoception in patients with hypertensive heart disease (HHD). Participants from two countries completed a facial emotion recognition test, and a subsample additionally underwent an interoception protocol based on a validated heartbeat detection task. HHD patients from both countries presented deficits in the recognition of overall and negative emotions. Moreover, interoceptive performance was impaired in the HHD group. In addition, a significant association between interoceptive performance and emotion recognition was observed in the control group, but this relation was abolished in the HHD group. All results survived after covariance with cognitive status measures, suggesting they were not biased by general cognitive deficits in the patients. Taken together, these findings suggest that emotional recognition alterations could represent a *sui generis* deficit in HHD, and that it may be partially explained by the disruption of mechanisms subserving the integration of neuro-visceral signals.

## Introduction

The intriguing relation between the heart and emotional processing has been gaining increasing attention in cognitive neuroscience, as best seen in recent theories of affect suggesting that interoceptive processing (i.e., the sensing of visceral feelings^1^) influences subjective emotional states an behaviors^2,3^. Alterations of the cardiovascular system can affect interoceptive processing^4,5^. However, the impact of cardiac abnormalities on emotional processing remains poorly understood. Although emotional alterations have been associated with high blood pressure in normotensive and subjects at risk of hypertension^6–8^, these studies lack precise diagnoses, fail to rule out key cognitive confounds, and offer no evaluation of interoceptive processing. The literature is thus deprived of key empirical evidence for neuro-visceral models of the phenomenon. Here, we present the first multicenter study assessing emotional recognition in essential hypertensive heart disease (HHD), accounting for the patients’ general cognitive status and assessing the links between emotion processing and cardiac interoception. In this way, our research contributes to understanding the relationship between emotional and interoceptive deficits in HHD, in particular, and its implications for theories of embodied emotional processing, in general^2,9–12^.

Today, the main challenge for characterizing the impact of hypertension of emotional processing lies in the shortcomings of available studies. Existing reports are undermined by sampling issues, including the recruitment of groups that are not matched for sociodemographic variables (e.g., age, gender and education^8^) or which prove ethnically biased (e.g., comprised only of Afro-American participants)^7^ or socio-economically restricted (e.g., including only low-income subjects)^7^, alongside investigations that directly lacked healthy controls^13^. Moreover, in most studies, HHD diagnosis has been rarely established on the basis of rigorous clinical examination^6–8,14,15^, which may mask potential links between emotional alterations and disease-specific abnormalities. In addition, most previous works have failed to include assessments of general cognitive functions^6–8,14,15^. This is a major caveat, too, since emotional processes are closely related to executive functions, memory, language, and otherwise general cognitive domains^16–22^, which, in turn, are typically affected in subjects with high blood pressure^23–28^. Without such data, the literature is moot on whether emotional disturbances in essential hypertension constitute primary deficits or secondary disruptions following from other general alterations triggered by high blood pressure.

Against this background, we performed the first multi-center study assessing emotional recognition in HHD patients diagnosed by specialized cardiologists in clinics from two countries. First, to control for possible cognitive differences between patients and controls^23–28^, and to account for the well-established role of language skills in emotion processing^21,22^, we administered an executive function battery and a highly sensitive cognitive screening tool including language measures (see details in “Cognitive assessment”, Table 1). Then, participants performed a validated emotion morphing task^29^. In addition, in Country-2, we capitalized on the opportunity to assess cardiac interoception, a key mediator of emotional processing in healthy subjects^3,30,31^ that is affected in hypertension^4,32^. This was done via a validated heartbeat detection task, known to be sensitive to interoceptive deficits in hypertensive subjects^4,32^. HHD affords a key model to this end, given its alterations in two interrelated mechanisms (emotional processing and interoception)^2,4,33,34^ and its high prevalence among heart diseases^35^, which allows recruiting a considerable sample that fulfills stringent inclusion and exclusion criteria. In light of previous reports, we predicted that HHD patients would exhibit emotion recognition deficits across countries, and that these would not be explained by global cognitive state or executive skills. Moreover, we hypothesized that emotional processing would be significantly associated with interoceptive performance in healthy controls, but that such a link would be abolished in the patients.

**Table 1:** Demographic, depression, and cognitive profiles of patients and controls.

Multicenter sample	HHD patients	Controls	F/(χ2)	*p*-value	ηp^2^
Gender	F = 36; M = 24	F = 37; M = 19	(0.50)	0.47	–
Age	61.00 (1.61)	58.85 (1.64)	(1,114) 0.86	0.35	0.01
Education	15.85 (0.45)	16.08 (0.46)	(1,114) 0.13	0.71	< 0.01
BDI	6.95 (1.03)	8.90 (1.01)	(1,96) 1.78	0.18	0.01
IFS	22.50 (0.38)	24.61 (0.39)	(1,112) 14.54	< 0.01*	0.11
ACE-R total	90.76 (0.53)	93.47 (0.55)	(1,112) 12.24	< 0.01*	0.09
ACE-R Verbal fluency	17.59 (6.45)	18.07 (6.67)	(1,114) 0.15	0.69	0.01
ACE-R language	25.09 (1.90)	25.06 (2.33)	(1,108) 0.06	0.93	0.00
ACE-R VLOM ratio	1.07 (0.18)	1.10 (0.17)	(1,108) 0.90	0.34	0.09
**Country-1**					
Gender	F = 23; M = 12	F = 20; M = 10	(0.006)	0.93	–
Age	57.20 (2.10)	52.43 (2.26)	(1,63) 2.37	0.12	0.03
Education	16.45 (3.09)	15.73 (3.32)	(1,63) 0.04	0.83	0.01
BDI	6.05 (1.11)	7.26 (1.20)	(1,63) 0.54	0.46	0.01
IFS	21.00 (0.47)	23.86 (0.44)	(1,63) 13.64	< 0.01*	0.17
ACE-R total	90.68 (0.57)	92.30 (0.61)	(1,63) 3.69	0.05*	0.06
ACE-R Verbal fluency	12.32 (1.29)	12.52 (1.38)	(1, 64) 0.33	0.56	0.00
ACE-R language	25.12 (2.14)	25.16 (1.09)	(1,64) 0.01	0.91	0.00
ACE-R VLOM ratio	0.97 (0.06)	0.99 (0.06)	(1,64) 1.29	0.26	0.02
**Country-2**					
Gender	F = 13; M = 12	F = 17; M = 9	(1.15)	0.28	–
Age	66.26 (1.63)	66.78 (1.73)	(1,47) 0.04	0.83	0.01
Education	16.50 (2.58)	15.00 (4.54)	(1,47) 2.20	0.114	0.03
BDI	11.35 (1.78)	9.50 (2.30)	(1,31) 0.40	0.53	0.01
IFS	23.95 (3.68)	25.26 (2.07)	(1,47) 2.70	0.13	0.04
ACE-R total	91.77 (0.89)	94.88 (0.83)	(1,47) 6.41	0.01*	0.12
ACE-R Verbal fluency	25.04 (1.42)	24.96 (3.20)	(1,47) 0.01	0.91	0.00
ACE-R language	25.05 (1.49)	24.90 (3.46)	(1,42) 0.03	0.86	00
ACE-R VLOM ratio	1.21 (0.19)	1.27 (0.13)	(1,42) 1.32	0.25	0.03

Asterisks (*) indicate significant differences.

*BDI* Beck’s Depression Inventory; *IFS* Ineco Frontal Screening battery; *ACE-R* Addenbrooke’s Cognitive Examination Revised.

## Methods

### Participants

This multicenter study comprised 116 participants (60 HHD patients and 56 healthy controls) recruited from two specialized centers (see Table 1 for demographic information). Using G*Power 3.1^36^, we performed a power estimation analysis for a one-way ANOVA, considering an alpha of α = 0.05, a power of 0.8^37^, and an effect size of η^2^ = 0.17 (as a median value between η^2^ = 0.08 and 0.27, based on previous reports for similar experimental tasks and samples^38–41^). Results indicated that a total sample size of 41, considering the two groups, was enough to reach the estimated effects. Our actual sample size (*n* = 116) reaches a power of 0.99. Participants were recruited from Faculty of Health, Santiago de Cali University (Country-1), and the INECO Foundation (Country-2). In both cases, participants were directly recruited from volunteer lists and through postings on relevant social media. The HHD samples were comprised of chronic outpatients from both institutions, each diagnosed with essential hypertension by expert cardiologists (R. S. and J. L.). In all cases, diagnosis was made following current revised criteria^42^ and guidelines from the American Heart Association^43^—for further details about clinical measures in each country, see Supplementary Table 1. The patients’ medical histories, provided by expert cardiologists, confirmed the absence of neurological, metabolic, or psychiatric antecedents (e.g., no history of heart attacks, controlled levels of cholesterol, controlled smoking history, no prior strokes, and absence of affective or personality disorders).

The HHD patients were matched with healthy controls in terms of age, gender, education, mood (as tapped through Beck’s Depression Inventory)^44^, and handedness (Table 1)—this pairing was achieved for each country separately and also for both together. None of the controls presented a history of drug abuse, psychiatric or neurologic disease, cognitive impairment or hypertension. All participants provided informed consent in accordance with the Declaration of Helsinki. The study was approved by the institutional ethics committees of both institutions, the Ethical Research Committee of the Faculty of Health, Santiago de Cali University (Country-1) and the Ethical Research Committee of INECO (Country-2).

### Multi-center analysis

#### Cognitive assessment

Cognitive abilities were examined with two screening measures. First, executive functions were assessed with the INECO Frontal Screening (IFS) battery^45^, a sensitive tool for neuropsychiatric assessment which taps eight relevant domains, including motor programming, conflict instructions, motor inhibitory control, working memory (numerical, verbal and spatial), abstraction capacity, and verbal inhibitory control. The maximum global score on the IFS is 30 points. Also, the subjects’ global cognitive status was established via the Addenbrooke’s Cognitive Examination Revised (ACE-R)^46^. This tool encompasses tests of five cognitive domains (attention/orientation, memory, language, verbal fluency, and visuospatial skills), yielding a maximum global score of 100. For a full description of these instruments, see Supplementary Table 2.

#### Facial emotion recognition

Participants performed a validated Emotional Morphing task^47^ based on the Pictures of Affect Series^48^. The task has proven sensitive for neurological^30,49–51^, psychiatric^52,53^, and cardiological^5^ conditions. It taps on six basic emotions: happiness, surprise, sadness, fear, anger, and disgust. Participants were instructed to recognize each emotion as fast as they could (watching a video with faces from 0 to 100% morphing) by pressing the ‘space’ key on a keyboard, and then they were requested to identify the emotion at hand from a word list showing the names of six basic emotions.

During the first stage, 48 faces were presented in videos that showed progressive changes in their shape and texture, with a morphing increment of 5%, from a neutral image (0%) to a full emotion face (100%) (for details, see the series of validation experiments in^47^ and recent adaptations^52,53^). These stimuli were randomly presented on a computer screen for a maximum of 6 s and subjects were allowed to interrupt the morphing processes if they were sure of what emotion had been presented (i.e., before 100% morphing). Upon the subject’s response or full elapsing of the six-second period, a list of basic emotion categories appeared, and subjects were required to choose the one that matched the face’s emotion. The task was run and analyzed by E-prime Version 2.0 and its toolbox E-studio and E-run.

For both the multicenter and single-country analyses, accuracy was indexed via three scores, namely: global score (i.e., for all emotions combined), in terms of valence (i.e., considering all negative emotions together [sadness, angry, disgust, and fear] and all positive emotions together [happiness and surprise]), and also considering each emotion type separately. Verbal labels were adapted to Spanish and previously validated with samples from both countries^50,52,53^.

#### Multicenter behavioral data analysis

To test whether the predicted impairments proved robust despite socio-cultural heterogeneity, we implemented two strategies: statistical analyses were first performed for the combination of samples from both countries, and then repeated for each country separately. Also, to maximize informativeness, we compared the groups’ emotion recognition performance via task via ANOVAs for (i) global scores, (ii) all negative emotions together, (iii) all positive emotions together, and (iv) each individual emotion separately. Gender was compared between groups with Pearson’s chi-squared (χ^2^) test. Age, education, and mood state data were assessed through ANOVAs. For the analysis of the facial emotion recognition task, we performed ANOVAs for the global, negative, and positive scores, and also for each individual emotion. Also, subjects deviating from the sample’s mean in at least 2 *SD*s were considered as outliers (as per Chauvenet’s criteria) to remove data points that may not reflect the psychophysiological processes targeted by the task, while increasing the power of the test to find truly significant results^52–54^, as shown in simulation studies^55–57^ (Supplementary Table 8). Furthermore, considering the cognitive deficits observed in the HHD group (see Table 1) and their potential influence on emotion processing^2,21,26,34^, we re-ran all emotion-recognition analyses using ANCOVAs, including the scores of the IFS and the ACE-R as covariates—all results are reported after this covariation (see “Results” section and Supplementary Tables 4 and 7. As a complementary strategy, we replicated our analyses upon removing subjects with scores indicative of cognitive deficits and explored correlations with potential confounding factors. Effect sizes were reported with partial eta squared (η_p_^2^).

### Complementary interoceptive assessment for Country-2

Given that interoceptive skills are pivotal for emotional processing^3,30,31,58^ and typically impaired in HHD^4,32^, we tested for associations between emotion recognition and interoception outcomes in Country-2, hypothesizing a positive correlation in controls and no such association in the patients. Correlations were performed by comparing scores in the heartbeat detection task (i.e., subject’ precision index: min 0.0–max 1.0) and global performance in emotion recognition (min 0.0–max 1.0).

#### Interoceptive performance: heartbeat detection task

Interoceptive performance was assessed through a modified version of a validated heartbeat detection task^29,59–64^, in which patients are asked to attend to their own heartbeats (see Fig. 2A and Supplementary Material C.). The task encompassed both a control and an interoceptive condition. In the control condition (a baseline measure of external monitoring skills), participants tapped a keyboard to follow binaurally presented heartbeats. This condition included two blocks of 2.5 min, featuring regularly timed and irregularly timed heartbeats, respectively. In the interoceptive condition (aimed to assess inner signal monitoring), participants tapped a key to follow their own heartbeats without any external cues. Each participant completed two 2.5-min blocks. The interoceptive condition provides a measure of interoceptive accuracy, namely, the subjects’ objective performance in following their own heartbeats^65^. While the interoceptive condition had participants follow a relatively uncertain signal (their own heartbeats), the control condition required following a regular and external recorded heartbeat that was easier to track and presented less uncertainty. Behaviorally, these are rather different tasks that measure different abilities but both are critical for the interpretation of the results from the heartbeat detection task^66,67^. In this way, the control condition allows checking that participants have preserved basic motor tracking abilities, thus ruling out domain-external factors as a possible source of potential between-group differences in interoceptive condition.

During all blocks, participants were requested to respond with their dominant hand, to keep their eyes on a fixation cross, and to avoid excessive blinking and moving while the latter remained on screen—for further details of the task, see^4^.

Interoceptive performance was analyzed for each subject through a precision index^59,61–63,68^ based on two scores, namely: correct answers and recorded heartbeats. The correct answers scores were calculated as the total number responses that matched each of the subject’s heartbeats. To estimate this match, every motor response is compared within a time window around every recorded heartbeat; if the tap input is temporally locked within any heartbeat, that response is considered as correct –the procedure to estimate the time window for each subject is detailed in^59^. On the other hand, recorded heartbeats refer to the total number heartbeats registered in each condition. Both scores were used to calculate behavioral accuracy, following this equation: 1 − (Recorded heartbeats − ∑ Correct answers)/(Recorded heartbeats). This precision index can vary between 0 and 1, with higher scores indicating only small differences between correct answers and recorded heartbeats, and, thus, better performance (see Supplementary Material C.).

#### Heartbeat detection task and correlations analyses

Interoceptive performance for Country-2 participants was compared between groups via one-way ANOVAs. Correlations between facial emotion recognition and interoceptive outcomes were examined through bi-variated Pearson’s correlations, with a significance threshold of *p* ≤ 0.05. Theoretically, given that the aim of the study was to analyze the interplay between a basic cardio-cognitive mechanism (interoception) involved in emotions processing in general, interoceptive performance was matched only with a global score of emotions recognition. Relative to single-emotion scores, the global score provides a greater number of trials and allows capturing overall performance regardless of the valence of each emotion. Moreover, given the limited range of trials for each emotion (only four trials per emotion are presented in the task), the global score gives a more fine-grained evaluation of the samples’ inter- and intra-group variability, which allows tackling heterogeneity across the participants’ performance.

Although the heartbeat detection task has been criticized in terms of internal validity^66^ and robustness^69,70^, these potential caveats have been challenged and several advantages have been noted^71–73^. Unlike Schandry’s classical heartbeat detection task, which overestimates the real perception of heartbeats considering all behavioral answers as accurate^66^, our task requires tracking every single heartbeat through motor tapping and our accuracy score considers both failed and accurate responses (see details in^62,74^). At the same time, performance was controlled by heart rate and heart rate variability, and the window selected to consider accuracy ratings was adjusted to subject’s heart rate (see details in Supplementary Material C.).

## Results

### Cognitive assessments

IFS outcomes revealed lower scores in patients for both the multicenter analysis (*p* < 0.01) and also when Country-1 (*p* < 0.01) was evaluated alone. Country-2 presented no between-group differences (*p* = 0.13). Similarly, Global ACE-R scores revealed significantly poorer cognitive status in patients than controls, when samples were combined for the multicenter approach (*p* < 0.01) and also when they were framed separately for Country-1 (*p* = 0.05) and Country-2 (*p* = 0.01). Null differences were found in language subscales for both the multicenter analysis and the single-country analyses. Statistical details are shown in Table 1 and Supplementary Table 3.

### Facial emotion recognition

#### Multicenter sample

**Figure 1:**
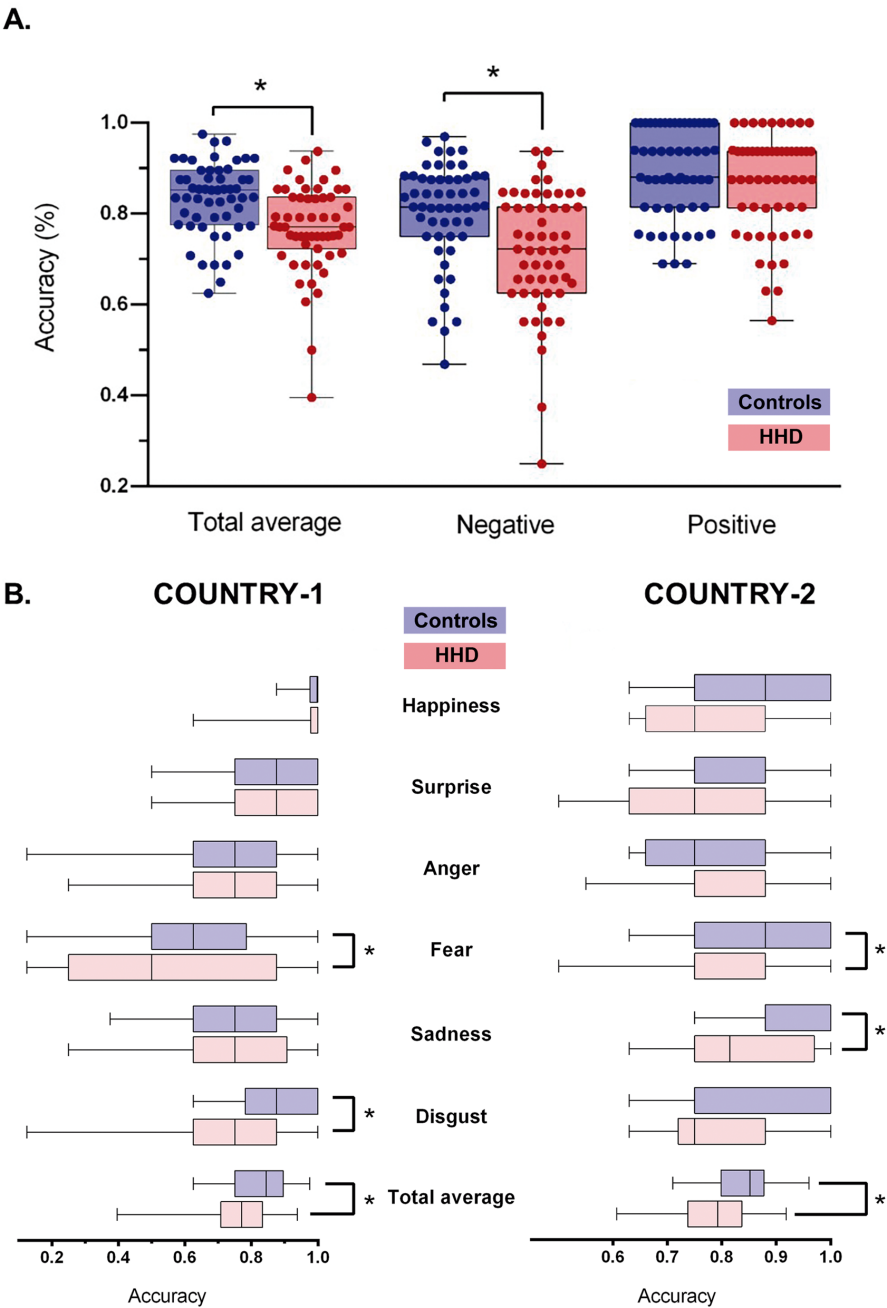
Facial emotion recognition results. (**A**) Multicenter results comparing patients and controls. Blue boxes represent controls and red boxes refer to HHD patients. The middle line in each box indicates mean. Whiskers indicate *SD*. Solid dots indicate each subject’s performance. Asterisks (*) indicate significant differences (*p* < 0.05) after analyses of covariance. (**B**) Single-country results. Violet boxes represent controls and pink boxes refer to HHD patients. The middle line in each box indicates mean. Whiskers indicate *SD*. Asterisks (*) indicate significant differences (*p* < 0.05) after analyses of covariance.

As shown by the accuracy index, patients were outperformed by controls when considering the total average of all emotions [*F* (1,108) = 12.01, *p* < 0.01, η_p_^2^ = 0.10] and negative emotions only [*F* (1,108) = 10.10, *p* < 0.01, η_p_^2^ = 0.08]—Fig. 1.A. No group differences were found for positive emotions [*F* (1,108) = 1.57, *p* = 0.21, η_p_^2^ = 0.01] or individual emotions—see Supplementary Table 6a. After selecting a cognitive unimpaired sample to replicate our main analysis (see criterion in the “Methods” section), we observed that comparison between the multicenter samples (i.e., global score, all negative emotions together, and all positive emotions together) remained intact (Supplementary Table 7). Finally, given that ‘surprise’ may be better classified as a negative emotion^75,76^, we repeated the analysis reallocating this emotion in the multicenter sample. Results remained the same (Supplementary Table 9).

#### Country-1

The accuracy index revealed significant poorer performance for patients than controls when considering the average of all emotions [*F* (1,63) = 4.46, *p* = 0.03, η_p_^2^ = 0.06], and when fear [*F* (1,60) = 3.01, *p* = 0.01, η_p_^2^ = 0.21] and disgust [*F* (1,61) = 15.71, *p* < 0.01, η_p_^2^ = 0.41] were analyzed in isolation—Fig. 1.B. No group differences were found for negative and positive scores or for isolated emotions (happiness, surprise, anger, and sadness)—see Supplementary Table 6a.

#### Country-2

The accuracy index revealed lower performance in patients than in controls when considering the average of all emotions [*F* (1,43) = 7.62, *p* < 0.01, η_p_^2^ = 0.15] and when fear [*F* (1,42) = 5.20, *p* = 0.02, η_p_^2^ = 0.11] and sadness [*F* (1,41) = 8.34, *p* < 0.01, η_p_^2^ = 0.17] were analyzed separately—Fig. 1.B. No group differences were found for negative and positive scores or for isolated emotions (happiness, surprise, anger, and disgust)—see Supplementary Table 6a.

### Complementary interoceptive assessment (Country-2 only)

**Figure 2:**
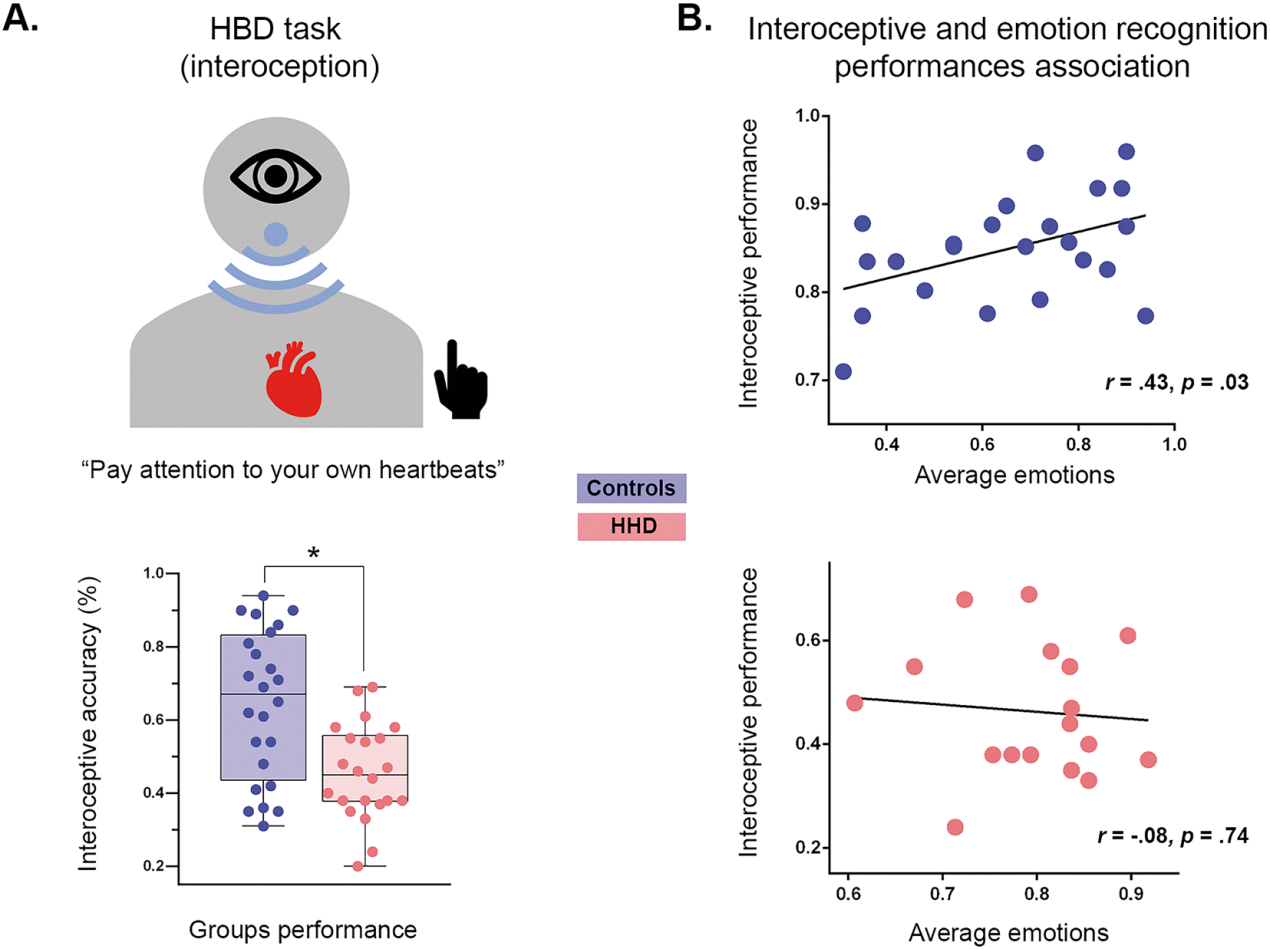
Associations between facial emotion recognition and interoceptive performance in Country-2. (**A**) (Top) Schematic representation of the heartbeat detection task. In the interoceptive condition, participants were asked to pay attention to their own heartbeats and tap a key in synchrony with them. (Bottom) Boxplot representation of interoceptive performance. The dots capture each subject’s performance; the horizontal lines inside the box represent the mean; the asterisks indicate statistical differences between groups (*p* = 0.05). (**B**) Scatterplots of Pearson’s correlations between interoceptive and emotion recognition outcomes for controls and HHD patients (Country-2). The Y axis represents interoceptive performance in interoceptive condition for each participant. The X axis represents the subjects’ mean score for overall facial emotion recognition.

Results from the heartbeat detection task revealed no between-group differences in the control condition [Controls: *M* = 0.77, *SD* = 0.14; HHD patients: *M* = 0.71, *SD* = 0.18 (*F* (1,40) = 1.19, *p* = 0.28, η_p_^2^ = 0.16)], alongside significantly poorer performance for patients than controls in the interoceptive condition [Controls: *M* = 0.64, *SD* = 0.20; HHD patients: *M* = 0.45, *SD* = 0.13 (*F* (1,41) = 13.29, *p* = 0.01, η_p_^2^ = 0.47)]—see Fig. 2, A-bottom.

Also, as a complementary analysis, we compared RTs between groups in each sets of emotions via ANCOVAs, considering the same factors and levels employed in the accuracy analyses. Results showed that patients were consistently slower than controls across emotions, countries, and their combinations (for details, see Supplementary Table 6b). Finally, to explore the relation between accuracy and RT results, we performed Pearson’s correlations between them in each group within the global score indexes. Whereas no significant association emerged in controls (*r* = 0.07, *p* = 0.59), a significant positive association was observed in HHD patients (*r* = 0.33, *p* = 0.01). This indicates that good outcomes in controls were achieved irrespective of processing time, whereas performance in patients was higher when they devoted more time to categorization decisions. For details, see Supplementary Fig. 6.

### Association between interoceptive performance and facial emotion recognition

A significant positive association was observed between interoceptive performance and the global emotion recognition score in controls (*r* = 0.43, p = 0.03), but not in HHD patients (*r* = − 0.08, p = 0.74)—Fig. 2B. Still, note that these results should be taken with caution as they were based on relatively small samples (24 controls and 17 HHD). To test the robustness of this result, we performed partial correlations controlling for cognitive outcomes. A significant association was found for controls (*r* = 0.46, *p* = 0.029) but not for HHD patients (*r* = − 0.11, *p* = 0.688). Additionally, given a different sample size in our independent variables, we generated the Student's *t*-distribution for testing correlations against each other^77,78^. Results confirmed that outcomes from the control and HHD samples were different (*Z* = 1.64, *p* = 0.05).

## Discussion

This is the first multicenter study to reveal systematic emotion recognition impairments in HHD. In both countries, patients exhibited overall emotion recognition impairments, with negative emotion deficits proving robust even in each country separately. Crucially, these disturbances emerged irrespective of the patients’ cognitive dysfunctions and they were unrelated to other potential confounding factors, such language outcomes^21,79,80^. Accordingly, they would seem to constitute primary (as opposed to epiphenomenal) disruptions. Moreover, complementary evidence from Country-2 revealed that associations between emotion recognition and cardiac interoception (but not other relevant domains) proved significantly positive for controls but were absent in HHD patients, suggesting a partial modulatory effect of viscero-sensing mechanisms in non-pathological emotion processing. Together, these results contribute to recent emotion theories^2,3^ that emphasize the importance of sensory integration mechanisms in the functioning of cognitive-emotional processes.

Affective dysregulation and deficits in emotion perception and recognition have been previously related to blood pressure alterations in normotensive individuals, subjects at risk for hypertension, and cardiological patients^5,6,8,13^. However, as noted at the outset, most of these works present several limitations, including imprecise assessments and diagnoses of HHD^6,7^ as well as scant or null efforts to control for cognitive impairments related to blood pressure alterations^8^. Here, we overcame these limitations by recruiting a representative multicenter sample with accurate clinical diagnosis while accounting for the potential impact of the patients’ domain-general functions. First, emotion recognition deficits were present across countries after controlling for cognitive status. These deficits were specific for negative emotions at large, and especially robust for disgust, sadness, and fear—the latter two emotions, in fact, were consistently affected in both countries. Interestingly, patients were consistently slower than controls, suggesting that they were not only less effective, but also less efficient at identifying emotions. In addition, accuracy was correlated with RTs in the patients but not in controls. This indicates that good outcomes in the latter were achieved irrespective of processing time, whereas performance in patients was higher when they devoted more time to categorization decisions. Although the overall deficit in emotional recognition mirrors previous results^6–8,13^, our study is the first to show that such an impairment may constitute a primary marker of hypertension, given that it is not explained by overall cognitive disturbances (see Supplementary Material A.). Our study also seems to be the first in reporting specific deficits in negative emotions for HHD patients, given that previous reports on this condition did not present or discuss outcomes in terms of contrastive emotional valances or particular emotions^6–8,13^.

Impaired negative emotion processing in HHD may be influenced by different mechanisms. For example, negative emotions are more dependent on working memory processes than positive ones^81,82^. However, our results remained the same after covarying for executive-function outcomes (IFS), including working memory tasks. Thus, the alterations observed in negative emotions might not be associated to deficits in this domain. On the other hand, in our multi-centric analysis, the global negative index involves an extensive list of basic emotions (such as fear, anger, sadness, and disgust for our main analysis; and including surprise in a complementary analysis [Supplementary Table 9]), whereas the global positive index is restricted to happiness and surprise (or even to happiness only, as shown in a complementary analysis [Supplementary Table 9]). Therefore, relative to positive emotions, negative stimuli include more individual items that might represent a potential mistake, hence decreasing the associated global score. Yet, when we evaluated each country separately, only individual negative emotions yielded impairments (fear, sadness and disgust), which suggests that the differences in the number of trials are not biasing our global results.

Finally, negative emotions generate greater arousal than positive ones^83–88^. Embodied models of emotional processing (as discussed below) suggest that the perception of bodily changes –mediated by interoceptive processes—is a key aspect that modulates subjective feelings and impacts on emotional processing^83–87^. Therefore, alterations in this domain, as shown by HHD patients here and in previous studies^4,32^, might have a larger impact on negative than positive emotions, which generate a less pronounced body arousal. In this way, regarding emotional processing, our results converge with previous evidence showing a prominence of negative emotion as core deficit within the emotional processing repertoire of HHD.

More particularly, results from Country-2 suggest that emotional deficits in HHD could be more related to interoceptive disturbances than to other domains. In fact, whereas emotion recognition and interoceptive outcomes were positively correlated in controls, they were impaired and non-correlated in the patients, even after accounting for the impact of cognitive outcomes and medication status (see “Results” section, Table 1, and Supplementary Material A.). These results align with previous studies showing direct links between interoception and emotion processing, in particular^12,30,89–96^, and social cognition deficits, in general^90,95,97^. More specifically, significant associations between interoceptive and emotional outcomes have been reported in a wide range of studies on healthy participants^68,91,98–100^, but no such correlations have emerged in research of psychiatric^60,63,101^, neurological^30,61,102^, and cardiovascular^5^ disorders presenting alterations in one (or both) of these domains. Moreover, interoception and emotion recognition share similar anatomical hubs^30,61^, including the insular, somatosensory, and anterior cingulate cortices^31,103–105^. Our findings are in line with embodied cognition proposals proposing that emotions are multifactorial constructs based, in part, on the perception of visceral information^2,21,33,106^. In this context, interoception has been proposed as a neurocognitive mechanism that modulates the subjective feeling and processing of emotions (and also other social cognition domains, such as decision making, empathy and theory of mind)^11,34^. As discussed above, this interaction between interoception and emotional processing is especially strong for negative emotions (e.g. fear, disgust, sadness, anger), given that they generate a greater physical arousal than positive ones^83–87^. Moreover, emotions may be modulated by visceral information and shaped by Bayesian inference mechanisms based on bodily inputs^3,11,107,108^. Those predicted emotional states could be altered when interoceptive pathways become impaired, as shown in HHD^4^, dampening^7^ the patients’ perception of their own emotions and the comprehension of emotions in others.

Concerning HHD, existing evidence shows a pattern of emotion emotional^6–8,13^ and interoceptive^4,32^ deficits. Some theoretical frameworks have been proposed to explain the relation between the alterations of these two domains in cardiovascular diseases^14^. For example, the *baroreflex hypothesis*^14,109^ indicates that the increment of baroreflex activity in the context of high blood pressure could reduce arousal and pain levels and as well as the recognition of negative emotions. On the other hand, the *dampening hypothesis*^6^ proposes an inverse relationship between resting blood pressure and emotion responsivity (e.g., pain perception, face emotion recognition)^6–8^. This relationship could be explained by the intimate association between blood pressure regulation in the central nervous system (CNS) and the CNS pathways mediating the expression of emotions, although the specific mechanisms involved are still unclear. In fact, recent evidence suggests that this association should be multifactorial (a basic biobehavioral mechanism, integrating emotional, stress-related, and blood pressure signals)^7^. Additionally, this hypothesis extends the dampening effect to pain perception, revealing the existence of a general viscero-central integration deficit. In sum, for these two theories, a series of metabolic and emotional conditions could generate more tolerance to negative emotions, leading to their faulty recognition in HHD^14^. Succinctly, these antecedents coalesce with our results to suggest that emotion recognition deficits in HHD might be associated with disruptions in interoceptive mechanisms.

Our results also align with the view that allostasis is regulated by an internal model of bodily function^110^ based on previous experiences^111^. Emotions^112^ are considered conceptualizations (i.e. high-order domains or metacognition) of those internal states, linked to interoceptive processes^113^. Indeed, interoceptive theories^33,58,114–117^ propose that emotions are constructed by predictions based on (bottom-up) bodily inputs interacting with both internal and external milieu by anticipating (top-down) mental states, feelings, and memories, as well as external (including emotion-laden) stimuli.

Note that interoceptive sensations are usually experienced as lower-order dimensional inputs in affective life^110^. In HHD literature, essential hypertension is associated with baroceptors impairments in the carotid^6,7^, that is, the source of cardiac interoception inputsfor a review (see^118–120^). Then, after a series of relays in the brainstem, thalamus and insula, interoceptive information is used as permanent feedback to construct high-order feelings or emotions^2,11^.

For this model, HHD conditions introduce interferences in the “normal” flow of inner information, as chronic high blood pressure would, altered interoceptive sensations^74,99,116,119,121^ could disrupt internal predictions. Under normal conditions, when the internal model of the body (top-down) receives new inputs (e.g., an emotional face), chemical adjustments balance the internal and external milieus^2,113^. Conversely, when high blood pressure alters inner states, this adaptive capacity of the system is dampened^7^ due to the expansion of tolerance thresholds^6^, compromising emotional processing.

Beyond its contributions, this work presents limitations that could be tackled in future studies. First, as expected^122–127^, our patients exhibited lower cognitive outcomes tan controls. Although we accounted for this issue through complementary approaches (ANCOVAs, replication analyses with cognitively unimpaired subsamples, correlations with language measures), future studies should assess whether similar outcomes are obtained in HHD samples with and without cognitive deficits. Second, patients were medicated with antihypertensive drugs at the moment of testing in both countries (Supplementary Table 1), as is typical in chronic cases^128,129^. Although our decision to interrupt medication 48 h before testing has been reported as a strategy to minimize domain-external confounds^4^, future studies should aim for broader samples including both medicated patients and non-medicated patients. Third, another limitation of our study is that it hinges on the basic emotions model^48^. It remains uncertain whether similar deficits might be present when considering more complex emotions (such as shame, guilt, among others), although similar alterations in HHD constitute a certain possibility to be explored in future research. Also, given that facial emotion recognition tasks are restricted to visual stimuli representing only basic emotion categories, future studies should consider the role of language and other contextual cues (such as body language and prosody) during the process of recognition and assignment of responses. Indeed, these variables are known to modulate facial emotion recognition^21,130^. Furthermore, beyond testing for emotion-label words (e.g., sadness, happiness, fear), future works should examine whether HHD patients are also affected in their capacity to process emotion-laden words (e.g., -*death*, *birthday*, *rape*), given that this different type of word categories depend on different cognitive processes^79,131–134^. Fourth, also, we did not control the impact of potential language deficits of the HHD sample in emotion perception^20,22,135^ given that we did not have an specific full battery to measure this domain. Yet, we found no statistical relationship between the “Language” sub-score of the ACE and the ‘global score’ of emotion recognition (Supplementary Table 5). Future studies should include a more comprehensive task to evaluate this ability and corroborate our null findings regarding its association with emotion recognition in HHD patients. Fifth, our correlation results from Country-2 stemmed from a small sample (see “Results” section). Therefore, they should be taken as very preliminary findings until new studies replicate the present analyses. Sixth, future experimental designs could fruitfully extend our present protocol through the addition of tasks tapping other social cognition domains (e.g., empathy or theory of mind), especially in more ecological settings. Finally, the association between interoception and emotional recognition performance was based only on the analysis of the heartbeat detection task. Future research on this field should evaluate the role of other interoceptive dimensions in emotional processing, including awareness^104,136–138^, sensibility^65^, learning^61,139^, and metacognition^61^. Moreover, such extensions of our study could benefit from the inclusion of relevant neurophysiological measures, such as the heart-evoked potential^4^.

This is the first multicenter study to evaluate emotion recognition processes in HHD. Our findings suggest that alterations in this domain are an intrinsic deficit of this disease (irrespective of other cognitive impairments) and that they may be related to deficits in neurocardiac integration dynamics, as indexed by interoceptive disruptions. Our findings thus nurture the theoretical understanding of embodied mechanisms related to emotional processing in healthy subjects while providing critical insights about their alteration on HHD patients. Further efforts in this direction could contribute to improving the clinical evaluation of this deficit and eventually be tested as a critical marker for diagnosing the disease, tracking its evolution, and assessing the patients’ response to treatment.

## Supplementary information

Supplementary file1
