# Supplementary material for: Multicentric evidence of emotional impairments in hypertensive heart disease
Source: Sci Rep. 2020 Aug 24;10:14131. doi: 10.1038/s41598-020-70451-x (PMC7445248; doi:10.1038/s41598-020-70451-x)
Supplement: Supplementary file 1 — Supplementary file1 [file 41598_2020_70451_MOESM1_ESM.docx]

**Multicentric evidence of emotional impairments in hypertensive heart disease**

**Adrián Yoris^1,2^, Agustina Legaz^2,3^, Sofía Abrevaya^1,2^, Sofía Alarco^1^, Jéssica López Peláez^4^, Ramiro Sánchez^5^, Adolfo M. García^2,3,6,7,8^, Agustín Ibáñez^2,3,8,9,10^, Lucas Sedeño^2*^**

^1^ Institute of Cognitive and Translational Neuroscience (INCYT), INECO Foundation, Favaloro University, Buenos Aires, Argentina

^2^ National Scientific and Technical Research Council (CONICET), Buenos Aires, Argentina

^3^ Universidad de San Andrés, Buenos Aires, Argentina

^4^ Faculty of Health, Santiago de Cali University, Cali, Colombia

^5^ Metabolic and Arterial Hypertension Unit, Favaloro Foundation Hospital, Buenos Aires, Argentina

^6^ Faculty of Education, National University of Cuyo (UNCuyo), Mendoza, Argentina

^7^ Departamento de Lingüística y Literatura, Facultad de Humanidades, Universidad de Santiago de Chile, Santiago, Chile

^8^ Global Brain Health Institute, University of California, San Francisco, United States

^9^ Universidad Autónoma del Caribe, Barranquilla, Colombia

^10^ Center for Social and Cognitive Neuroscience (CSCN), School of Psychology, Universidad Adolfo Ibáñez, Santiago, Chile

**^*^ Corresponding author:** Lucas Sedeño**.** Pacheco de Melo 1860, C1126AAB, Buenos Aires, Argentina**. Phone and fax:** +54 (11) 4807-4748**. E-mail address:** lucas.sedeno@gmail.com

**Supplementary Material**

1. **Additional clinical and cognitive data**

**Supplementary Table 1. Complementary clinical measurements in HHD samples**

| Cardiovascular assessment | HHD group | Control sample | Statistics |
| --- | --- | --- | --- |
| **Country-1** |  |  |  |
| Systolic OBP | 124.33 | 115.53 | *p* = .01* |
| Diastolic OBP | 89.03 | 70.04 | *p* = .01* |
| Years since diagnosis | 1 to 10 years | -- | -- |
| Medication** (%) |  | 0 | -- |
| Sertraline | 4.34 | 0 | -- |
| Fluoxetine | 4.34 | 0 | -- |
| **Country-2** |  |  |  |
| ABPM (systolic 24 h) | 131.33 (10-72) | 112.72 (15.67) | *p* = .02* |
| OBP systolic | 148.47 (18.80) | 134.04 (14.99) | *p* = .01* |
| OBP diastolic | 81.82 (8.91) | 75.78 (9.01) | *p* = .03* |
| Time Diagnosis | 0.1 to 8 years |  |  |
| Medication*** (%) |  |  | -- |
| Hydrochlorothiazide | 20 | 0 | -- |
| Enalapril | 52 | 0 | -- |
| Nifedipine | 18 | 0 | -- |

Single asterisks (*) indicate significant differences; Double asterisks (**) indicate significant differences for mood and anxiety symptoms; Triple asterisks (***) indicate significant differences for hypertensive treatment. OBP: office blood pressure; ABPM: ambulatory blood pressure monitor.

**Supplementary Table 2.** Cognitive subdomains measured by the IFS and the ACE-R.

|  | |
| --- | --- |
| INECO Frontal Screening (IFS) battery ^[1]^ | |
| Motor programming | This subtest asks the patient to perform the Luria series, “fist, edge, palm” by initially copying the administrator, and by subsequently doing the series on his or her own then by repeating the series six times alone. If subjects achieved six consecutive series by themselves, the score was 3, if they achieved at least three consecutive series on their own, the score was 2; if they failed at achieving at least three consecutive series alone, but achieved three when copying the examiner, the score was 1; otherwise the score was 0. |
| Conflict instructions | Subjects were asked to hit the table once when the administrator hit it twice, or to hit the table twice when the administrator hit it only once. To ensure the subject had clearly understood the task, a practice trial was performed in which the administrator first hit the table once, three  times in succession, and then twice, three more times.  If subjects made no errors, the score was 3; if they made one or two  errors, the score was 2; for more than two errors, the score was 1, unless the subject copied the examiner at least four consecutive times, in which case the score was 0. |
| Motor inhibitory control | This task was administered immediately after test 2. Subjects were told that now, when the test administrator hit the table once, they should hit it once as well, but when the examiner hit twice, they should do nothing. If subjects made no errors, the score was 3; for one or two  errors the score was 2; for more than two errors the score was 1, unless the subject copied the examiner at least four consecutive times, in which case the score was 0. |
| Working memory | **Backward Digit Span:** subjects were asked to repeat a progressively lengthening string of digits in the reverse order. Two trials were given at each successive list length, beginning at two and continuing to a maximum of seven. If subjects passed either trial at a given list length, then the next length was administered. The score was the number of lengths at which the subject passed either trial, maximum 6.  **Verbal working memory:** The patient was asked to list the months of the year backward, starting with December. If subjects made no errors, the score was 2; for one error, the score was 1; otherwise the score was 0.  **Spatial Working Memory:**  the examiner presented the subject with four cubes and pointed at them in a given sequence. The subject was asked to repeat the sequence in reverse order. There were four trials, with sequences of two, three, four, and five cubes respectively. Score was number of correctly completed sequences. |
| Abstraction capacity | In this task, three proverbs were read to the subjects and they were asked to explain their meaning. For each proverb a score of 1 was given when the subject gave an adequate explanation, and a score of 0.5 for a correct example. Otherwise the score was 0. |
| Verbal inhibitory control | Inspired by the Hayling test, this task measures a subject’s capacity to inhibit an expected response. Materials were six sentences, each missing the last word and constructed to strongly constrain what it should be. In the first part (three sentences), subjects were read each  sentence and asked to complete it correctly, as quickly as possible. In the second part (remaining three sentences), subjects were asked for a completion that was syntactically correct but unrelated to the sentence in meaning. Only the second part was scored. For each sentence, a  score of 2 was given for a word unrelated to the sentence, a score of 1 for a word semantically related to the expected completion, and a score of 0 for the expected word itself. |
| Addenbrooke’s Cognitive Examination Revised (ACE-R) ^[2]^ | |
| Attention/orientation | Attention/orientation is tested by asking the patient for the date including the season and the current location; repeating back three simple words, and serial subtraction (e.g. subtracting seven from 100 and then continue subtracting seven away from each new number). |
| Memory | Memory is tested by asking the patient to recall the three words previously repeated; memorizing and recalling a fictional name and address; and recalling widely known historical facts. The memory section is split into five sections scattered throughout the tests. |
| Verbal Fluency | Fluency is tested by asking the patient to say as many words as they can think of starting with the letter (P) within one minute, and naming as many animals as they can think of in one minute. |
| Language | Language is tested by asking the patient to complete a set of sequenced physical commands using a pencil and piece of paper such as "place the paper on top of the pencil", to write two grammatically-complete sentences; to repeat several polysyllabic words and two short proverbs; to name the objects shown in 12 line drawings, and answer contextual questions about some of the objects; and to read aloud five commonly-mispronounced words. Language involves ascribing meaning to words and statements so this section consists of simple directions that may involve movements, such as the example of placing the paper on top of the pencil, to see how well they apply to mean. This section is the longest consisting of seven separate parts |
| Visuospatial abilities | Visuospatial abilities are tested by asking the patient to copy two diagrams; to draw a clock face with the hands set at a specified time; to count sets of dots, and to recognize four letters which are partially obscured. |

**Supplementary Table 3.** Multicenter and sub-samples cognitive status.

| **Cognitive functions** | HHD group | | Control sample | | Statistics | | |  |  |
| --- | --- | --- | --- | --- | --- | --- | --- | --- | --- |
| **Multicenter sample** |  | |  | |  | | |  |  |
| ACE-R Attention/orientation | 17.62 (.83) | | 17.79 (.53) | | *p* = .21, *ηp^2^* = 01 | | |  |  |
| ACE-R Memory | 20.86 (2.59) | | 22.29 (2.83) | | *p* = .01*, *ηp^2^* = 06 | | |  |  |
| ACE-R Visuospatial | 15.24 (1.17) | | 15.30 (1.60) | | *p* = .81, *ηp^2^* = 00. | | |  |  |
| **Country-1** |  | |  | |  | | |  |  |
| ACE-R Attention/orientation | 17.94 (.23) | | 18.00 (.00) | | *p* = .17, *ηp^2^* = .02 | | |  |  |
| ACE-R Memory | 19.85 (1.25) | | 20.90 (2.61) | | *p* = .04*, *ηp^2^* = .06 | | |  |  |
| ACE-R Visuospatial | 15.56 (.70) | | 15.55 (.81) | | *p* = .95, *ηp^2^* = .00 | | |  |  |
| **Country-2** |  | |  | |  | | |  |  |
| ACE-R Attention/orientation | 17.17 (1.12) | | 17.52 (.71) | | *p* = .19, *ηp^2^* = .03 | | |  |  |
| ACE-R Memory | 22.29 (3.27) | | 24.00 (2.12) | | *p* = .03*, *ηp^2^* = .09 | | |  |  |
| ACE-R Visuospatial | 14.79 (1.53) | | 15.00 (2.21) | | *p* = .70, *ηp^2^* = .00 | | |  |  |
| **Multicenter sample** | |  | |  | | |  | | |
| ISF global score | | 24.33 (3.39) | | 26.16 (2.21) | | *p* = .01*, *ηp^2^* = .09 | | |  |
| IFS Motor programming | | 2.48 (.68) | | 2.55 (.74) | | | *p* = .59, *ηp^2^* = .09 | | |
| IFS Conflict instructions | | 2.83 (.45) | | 2.96 (.19) | | | *p* = .37, *ηp^2^* = .07 | | |
| IFS Motor inhibitory control | | 2.60 (.77) | | 2.72 (.56) | | | *p* = .01*, *ηp^2^* = .09 | | |
| IFS Working memory | | 2.50 (.66) | | 2.79 (.54) | | | *p* = .01*, *ηp^2^* = .04 | | |
| IFS Abstraction capacity | | 2.35 (.72) | | 2.61 (.59) | | | *p* = .05, *ηp^2^* = .03 | | |
| ISF Verbal inhibitory control | | 4.98 (1.18) | | 5.27 (.87) | | | *p* = .14, *ηp^2^* = .02 | | |
| **Country-1** | |  | |  | | |  | | |
| ISF global score | | 24.59 (3.20) | | 26.71 (2.14) | | | *p* = .00*, *ηp^2^* = .13 | | |
| IFS Motor programming | | 2.20 (.72) | | 2.25 (.85) | | | *p* = .79, *ηp^2^* = .00 | | |
| IFS Conflict instructions | | 2.73 (.56) | | 3.00 (.00) | | | *p* = .01*, *ηp^2^* = .09 | | |
| IFS Motor inhibitory control | | 2.73 (.56) | | 2.61 (.66) | | | *p* = .42, *ηp^2^* = .01 | | |
| IFS Working memory | | 2.23 (.52) | | 2.75 (.53) | | | *p* = .00*, *ηp^2^* = .19 | | |
| IFS Abstraction capacity | | 2.32 (.63) | | 2.41 (.67) | | | *p* = .55, *ηp^2^* = .00 | | |
| ISF Verbal inhibitory control | | 4.91 (1.31) | | 5.16 (.93) | | | *p* = .38, *ηp^2^* = .01 | | |
| **Country-2** | |  | |  | | |  | | |
| ISF global score | | 23.96 (3.68) | | 25.48 (2.14) | | | *p* = .08, *ηp^2^* = .06 | | |
| IFS Motor programming | | 2.90 (.29) | | 2.95 (.20) | | | *p* = .53, *ηp^2^* = .00 | | |
| IFS Conflict instructions | | 3.00 (.00) | | 2.91 (.28) | | | *p* = .16, *ηp^2^* = .04 | | |
| IFS Motor inhibitory control | | 2.40 (1.00) | | 2.86 (.34) | | | *p* = .04*, *ηp^2^* = .09 | | |
| IFS Working memory | | 2.90 (.67) | | 2.84 (.57) | | | *p* = .71, *ηp^2^* = .00 | | |
| IFS Abstraction capacity | | 2.40 (.85) | | 2.86 (.34) | | | *p* = .02*, *ηp^2^* = .11 | | |
| ISF Verbal inhibitory control | | 5.09 (.97) | | 5.43 (.78) | | | *p* = .19, *ηp^2^* = .03 | | |

^ACE-R global score, Verbal fluency and Language subscales were included in main results (Table 1). Asterisks (*) indicate significant differences

**Supplementary Table 4.** Frequency of HHD patients performing below the cut-off in the ACE-R and the IFS battery

| **Cognitive performance** | HHD group | % |
| --- | --- | --- |
| **ACE’s cutoff = 85/100** ^[3]^ |  |  |
| Multicenter sample | 3 | *2.77%* |
| Country-1 | 1 | *3.22%* |
| Country-2 | 2 | *7.69%* |
| **IFS’s cutoff 19/30 ^[1]^** |  |  |
| Multicenter sample | 3 | *2.77%* |
| Country-1 | 1 | *3.22%* |
| Country-2 | 2 | *7.69%* |

Asterisks (*) indicate significant differences

**Supplementary Table 5.** Pearson’s correlation between the ‘Language’ sub-score of the ACE-R and the ‘global score’ of emotion recognition

|  | N | *P-value** | *R* |
| --- | --- | --- | --- |
| Multicenter sample |  |  |  |
| Healthy controls | 54 | .158 | -0.195 |
| HHD | 55 | .456 | -0.103 |
| Country-1 |  |  |  |
| Healthy controls | 24 | .255 | -0.242 |
| HHD | 20 | .507 | -0.158 |
| Country-2 |  |  |  |
| Healthy controls | 30 | .780 | -0.053 |
| HHD | 35 | .135 | 0.257 |

* Association analyses were performed using the Pearson’s correlation test. HHD: hypertensive heart disease patients.

1. **Additional emotion processing performance**

**Supplementary Table 6.a. Group performance for total average and basic emotions.**

**Multicenter results**

| **Basic emotions** | HHD group | Control sample | Statistics |
| --- | --- | --- | --- |
| Total average | .76 (.09) | .83 (.08) | *p* < .01*, *ηp^2^* = .10 |
| Negative emotions | .71 (.13) | .80 (.11) | *p* < .01*, *ηp^2^* = .08 |
| Positive emotions | .86 (.10) | .89 (.09) | *p* = .21, *ηp^2^* = .01 |
| Happiness | .91 (.13) | .93 (.11) | *p* = .46, *ηp^2^* = .00 |
| Surprise | .82 (.13) | .85 (.12) | *p* = .16, *ηp^2^* = .01 |
| Anger | .74 (.19) | .78 (.17) | *p* = .36, *ηp^2^* = .01 |
| Fear | .61 (.19) | .74 (.22) | *p* <.01*, *ηp^2^* = .07 |
| Sadness | .76 (.22) | .81 (.16) | *p* = .18, *ηp^2^* = .02 |
| Disgust | .75 (.15) | .84 (.15) | *p* <.01*, *ηp^2^* = .12 |

**Country-1**

| **Basic emotions** | HHD group | Control | Statistics |
| --- | --- | --- | --- |
| Happiness | .97 (.07) | .99 (.03) | *p* = .35, *ηp^2^* = .09 |
| Surprise | .87 (.11) | .90 (.13) | *p* = .27, *ηp^2^* = .12 |
| Anger | .73 (.21) | .76 (.21) | *p* = .56, *ηp^2^* = .07 |
| Fear | .51 (.04) | .63 (.05) | *p* = .01, *ηp^2^* = .21* |
| Sadness | .72 (.03) | .73 (.03) | *p* = .73, *ηp^2^* = .00 |
| Disgust | .72 (.02) | .88 (.03) | *p* < .01, *ηp^2^* = .20* |
| Total average | .77 (.08) | .81 (.09) | *p* = .02, *ηp^2^* = .09* |

**Country-2**

| **Basic emotions** | HHD group | Control | Statistics |
| --- | --- | --- | --- |
| Happiness | .79 (.12) | .84 (.13) | *p* = .21, *ηp^2^* = .19 |
| Surprise | .73 (.14) | .80 (.10) | *p* = .10, *ηp^2^* = .27 |
| Anger | .77 (.16) | .80 (.13) | *p* = .55, *ηp^2^* = .10 |
| Fear | .78 (.03) | .88 (.02) | *p* = .02, *ηp^2^* = .11* |
| Sadness | .83 (.02) | .93 (.02) | *p* < .01, *ηp^2^* = .17* |
| Disgust | .78 (.02) | .83 (.02) | *p* = .26, *ηp^2^* = .02 |
| Total Average | .78 (.07) | .84 (.05) | *p* < .01, *ηp^2^* = .17* |

Asterisks (*) indicate significant differences.

**Supplementary Table 6.b. Reaction times in the emotion recognition task.**

Multicenter results

| Basic emotions | HHD group | Control sample | Statistics |
| --- | --- | --- | --- |
| Total average | 11.06 (2.19) | 10.53 (2.50) | *p* = .57, *ηp^2^* = .00 |
| Negative emotions | 11.36 (2.62) | 10.82 (2.25) | *p* = .33, *ηp^2^* = .00 |
| Positive emotions | 10.45 (2.55) | 9.97 (2.40) | *p* = .76, *ηp^2^* = .00 |
| Happiness | 10.21 (2.94) | 9.93 (3.18) | *p* = .18, *ηp^2^* = .01 |
| Surprise | 10.69 (2.87) | 10.01 (2.21) | *p* = .32, *ηp^2^* = .00 |
| Anger | 11.45 (2.84) | 11.13 (2.53) | *p* = .72, *ηp^2^* = .00 |
| Fear | 11.18 (2.72) | 10.85 (2.36) | *p* = .55, *ηp^2^* = .00 |
| Sadness | 11.36 (2.99) | 10.79 (2.59) | *p* = .47, *ηp^2^* = .00 |
| Disgust | 11.44 (2.55) | 10.50 (2.22) | *p* = .03*, *ηp^2^* = .04 |

Country-1

| Basic emotions | HHD group | Control | Statistics |
| --- | --- | --- | --- |
| Happiness | 9.50 (2.53) | 9.13 (1.51) | *p* = .26, *ηp^2^* = .02 |
| Surprise | 11.29 (2.72) | 10.27 (1.98) | *p* = .75, *ηp^2^* = .00 |
| Anger | 12.14 (3.02) | 11.62 (2.28) | *p* = .10, *ηp^2^* = .04 |
| Fear | 11.71 (2.77) | 10.98 (1.95) | *p* = .59, *ηp^2^* = .00 |
| Sadness | 12.15 (3.08) | 11.47 (2.27) | *p* = .06, *ηp^2^* = .05 |
| Disgust | 12.21 (2.46) | 10.82 (2.18) | *p* = .75, *ηp^2^* = .00 |
| Total average | 11.50 (2.58) | 10.71 (1.79) | *p* = .33, *ηp^2^* = .01 |

Country-2

| Basic emotions | HHD group | Control | Statistics |
| --- | --- | --- | --- |
| Happiness | 11.42 (3.24) | 10.91 (4.30) | *p* = .60, *ηp^2^* = .00 |
| Surprise | 9.67 (2.90) | 9.69 (2.62) | *p* = .53, *ηp^2^* = .00 |
| Anger | 10.29 (2.09) | 10.29 (2.74) | *p* = .31, *ηp^2^* = .01 |
| Fear | 10.28 (2.43) | 10.69 (2.82) | *p* = .59, *ηp^2^* = .00 |
| Sadness | 10.02 (2.34) | 9.94 (2.75) | *p* = .15, *ηp^2^* = .04 |
| Disgust | 10.13 (2.19) | 10.10 (2.25) | *p* = .17, *ηp^2^* = .04 |
| Total Average | 10.30 (2.41) | 10.31 (2.62) | *p* = .34, *ηp^2^* = .02 |

RT expressed in seconds

Asterisks (*) indicate significant differences.

**Supplementary Figure 6. RT/accuracy associations in global scores of emotion recognition task.**


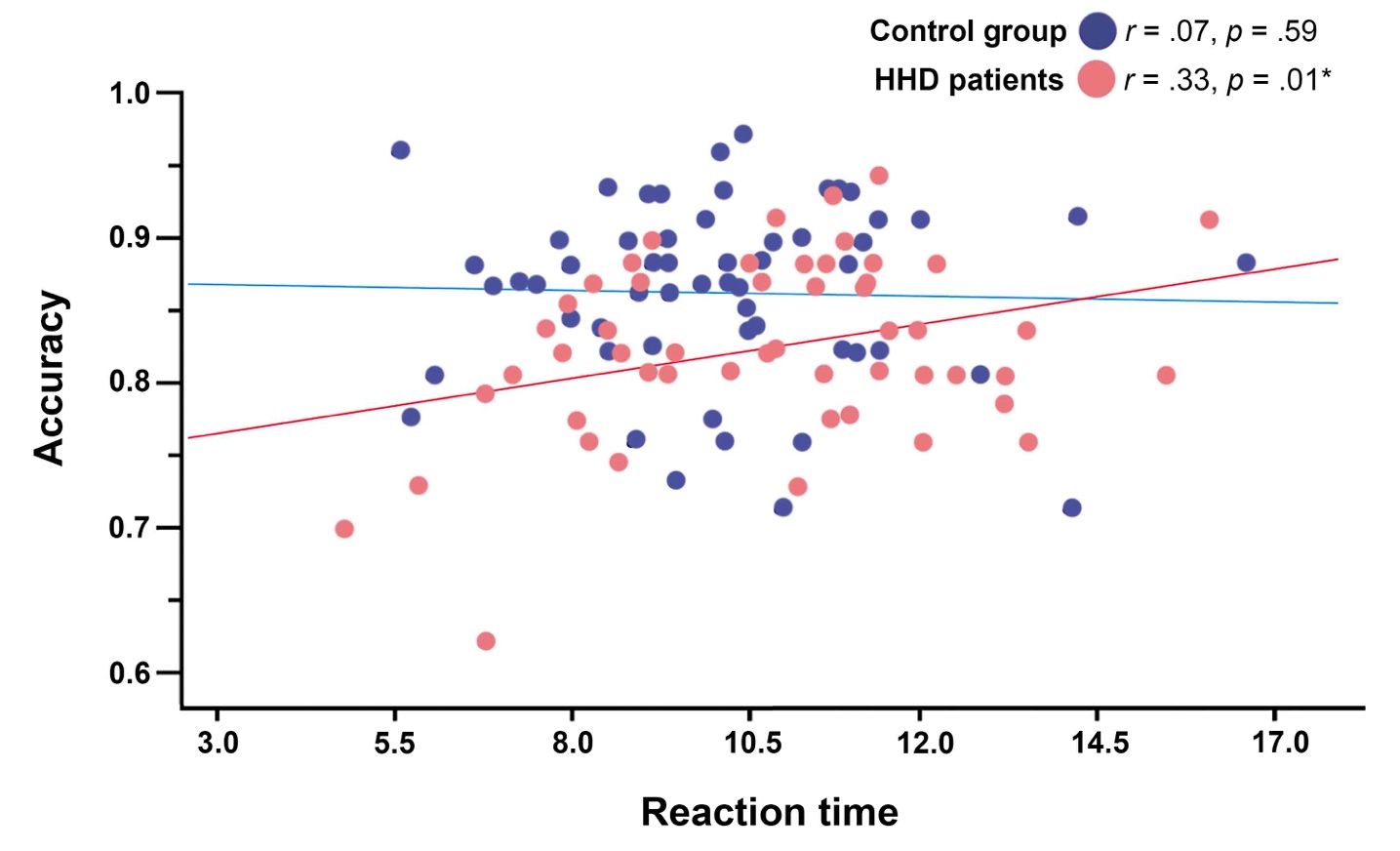


**Supplementary Figure 6.** Associations between accuracy and reaction time in HHD patients and controls from the multicenter sample for global score indexes. Red dots represent HHD patients and blue dots represent control subjects. Whereas no significant association emerged in controls, a significant positive association was observed in HHD patients. This indicates that good outcomes in controls were achieved irrespective of processing time, whereas performance in patients was higher when they devoted more time to categorization decisions. HHD: hypertensive disease patients.

**Supplementary Table 7.** Reanalysis of main results after cutoff criteria (ISF >= 19; ACE-R >= 85).

| **Multicenter** | HHD group | Control sample | Statistics |
| --- | --- | --- | --- |
| Total average | .77 (.08) | .82 (.04) | *p* < .01*, *ηp^2^* = .08 |
| Negative emotions | .72 (.13) | .79 (.11) | *p* < .01*, *ηp^2^* = .08 |
| Positive emotions | .86 (.11) | .89 (.09) | *p* = .24, *ηp^2^* = .01 |

Asterisks (*) indicate significant differences

**Supplementary Table 8.** Outliers rejected after 2SD threshold from the mean.

| Subgroup | Happiness | Disgust | Anger | Fear | Surprise | Sadness | Global score | Negative | Positive |
| --- | --- | --- | --- | --- | --- | --- | --- | --- | --- |
| Controls Country-2 | 2 | 0 | 1 | 1 | 1 | 1 | 0 | 0 | 0 |
| HHD Country-2 | 0 | 0 | 0 | 0 | 0 | 0 | 0 | 0 | 0 |
| Controls Country-1 | 0 | 0 | 0 | 0 | 0 | 0 | 0 | 0 | 0 |
| HHD Country-1 | 0 | 0 | 0 | 0 | 0 | 0 | 0 | 0 | 0 |
| **Total rejected** | **2** | **0** | **1** | **1** | **1** | **1** | **0** | **0** | **0** |

**Supplementary Table 9. Facial emotion recognition in the multicenter sample.**

| **Multicenter sample** | **HHD patients** | **Controls** | **F** | ***p*-value** | **ηp^2^** |
| --- | --- | --- | --- | --- | --- |
| Negative valence | .73 (.11) | .81 (.09) | (1/108) 10.293 | *<.01** | 0.33 |
| Positive valence | .91 (.12) | .92 (.11) | (1/106) .53 | *.46* | .04 |

* indicates significative differences. Negative valence included anger, fear, sadness, disgust, and surprise and positive valence only included happiness.

1. **Additional Heartbeat Detection task (HBD) description**

Interoceptive performance was assessed through a modified version of a validated HBD task ^[4-14]^, in which participants were asked to attend to their heartbeats. This task has been successfully implemented to measure *cardiac interoceptive performance* in neurological ^[11,15,16]^, psychiatric ^[6,9,17]^, and cardiological ^[18,19]^ conditions, and even in an extensive healthy sample^[12]^.

**Task procedure:** The HBD task encompassed both a control and an interoceptive condition. The **control condition** provides a measure assessing the subjects’ capacity to attend to external stimuli. Participants were binaurally presented with audio of a recorded heartbeat (digitally constructed from an actual electrocardiogram record of a researcher) and were instructed to follow them by pressing a key with their dominant hand. This condition encompassed two blocks of 2.5 ​min each. In the first block, recorded heartbeats were presented at a constant and regular frequency (60 bpm), while in the second block, recorded heartbeats were manipulated to have the same overall frequency (60 bpm) but at irregular intervals. Both blocks of the control condition were presented for all participants in the same order, before starting with the interoceptive condition.

**The interoceptive condition** was designed to provide an objective measure of the subjects’ ability to track their heartbeats ^[20]^. In two blocks of 2.5 min, participants were asked to tap a key with their dominant hand following their own heartbeats. They were advised do not to use any external signals. At the same time subjects performed the HBD task, we recorded the electrocardiographic signals to register the heartbeats alongside motor responses over time.

**Accuracy time-window formula:** Based on previous works of our group ^[4,6,11,12,16-19]^, we calculated performance on the HBD task considering the R-wave-EEG signal. To measure participants’ ability to follow their heartbeats, we calculated a **precision index** based on the following intermediate outputs:

**Total correct answers:** this index shows the total number of responses that matched each of the subject’s heartbeats. Every motor response is compared within a specific time window around every recorded heartbeat; if the tap input is temporally locked within a time window for any heartbeat, that response is considered as correct (the time window is determined by the subjects’ heart rate: between 125 ms before and 750 ms after the beat, for a heart rate less than 69.76; between 100 ms before and 600 ms after, for heart rates between 69.75 and 94.25; and between 75 ms before and 400 ms after, for heart rates higher than 94.25). The output of the index is the total sum of all responses that fulfill this temporal criterion.

**Recorded heartbeats:** this index refers to the total amount of heartbeats recorded in each condition.

The **precision index** was obtained through a modified equation of the one proposed by Schandry for his mental heartbeat tracking method ^[21]^. The accuracy equation we used is:

**1 - (Recorded heartbeats – ∑ Correct Answers)**

**______________________________________**

**Recorded heartbeats**

This precision index can vary between 0 and 1, with higher scores indicating only small differences between correct answers and recorded heartbeats, and, thus, better performance.

**Task duration**: the whole task occurs in 10 minutes approximately, contemplating the automatic sum of 2.5-minute by each block. A break before blocks were used to present the task instruction.

**Data analysis software:** ECG data was recorded via a Biosemi system. Two external electrodes were included to record ECG data. Heart peaks were imported beat-to-beat RR interval data from the ECG using the *Matlab* platform and paired with subject responses.

**References**

1 Torralva, T., Roca, M., Gleichgerrcht, E., Lopez, P. & Manes, F. INECO Frontal Screening (IFS): a brief, sensitive, and specific tool to assess executive functions in dementia. *J Int Neuropsychol Soc* **15**, 777-786, doi:S1355617709990415 [pii]

10.1017/S1355617709990415 (2009).

2 Torralva, T. *et al.* Validation of the Spanish Version of the Addenbrooke's Cognitive Examination-Revised (ACE-R). *Neurologia* **26**, 351-356, doi:10.1016/j.nrl.2010.10.013 (2011).

3 Hsieh, S., Schubert, S., Hoon, C., Mioshi, E. & Hodges, J. R. Validation of the Addenbrooke's Cognitive Examination III in frontotemporal dementia and Alzheimer's disease. *Dement Geriatr Cogn Disord* **36**, 242-250, doi:10.1159/000351671 (2013).

4 Melloni, M. *et al.* Preliminary evidence about the effects of meditation on interoceptive sensitivity and social cognition. *Behavioral and brain functions : BBF* **9**, 47, doi:10.1186/1744-9081-9-47 (2013).

5 Couto, B. *et al.* The man who feels two hearts: the different pathways of interoception. *Social cognitive and affective neuroscience* **9.9**, 1253-1260, doi:10.1093/scan/nst108 (2013).

6 Sedeno, L. *et al.* How do you feel when you can't feel your body? Interoception, functional connectivity and emotional processing in depersonalization-derealization disorder. *PloS one* **9**, e98769, doi:10.1371/journal.pone.0098769 (2014).

7 Garcia-Cordero, I. *et al.* Feeling, learning from and being aware of inner states: interoceptive dimensions in neurodegeneration and stroke. *Philosophical transactions of the Royal Society of London. Series B, Biological sciences* **371**, doi:10.1098/rstb.2016.0006 (2016).

8 Yoris, A. *et al.* The roles of interoceptive sensitivity and metacognitive interoception in panic. *Behav Brain Funct* **11**, 14, doi:10.1186/s12993-015-0058-8 (2015).

9 Yoris, A. *et al.* The inner world of overactive monitoring: neural markers of interoception in obsessive–compulsive disorder. *Psychol Med*, 1-14 (2017).

10 de la Fuente, A. *et al.* Multimodal neurocognitive markers of interoceptive tuning in smoked cocaine. *Neuropsychopharmacology* **44**, 1425-1434, doi:10.1038/s41386-019-0370-3 (2019).

11 Salamone, P. C. *et al.* Altered neural signatures of interoception in multiple sclerosis. *Human brain mapping* **39**, 4743-4754, doi:10.1002/hbm.24319 (2018).

12 Fittipaldi, S. *et al.* A multidimensional and multi-feature framework for cardiac interoception. *NeuroImage* **212**, 116677, doi:10.1016/j.neuroimage.2020.116677 (2020).

13 Garcia-Cordero, I. *et al.* Attention, in and Out: Scalp-Level and Intracranial EEG Correlates of Interoception and Exteroception. *Frontiers in neuroscience* **11**, 411, doi:10.3389/fnins.2017.00411 (2017).

14 Canales-Johnson, A. *et al.* Auditory Feedback Differentially Modulates Behavioral and Neural Markers of Objective and Subjective Performance When Tapping to Your Heartbeat. *Cereb Cortex* **25**, 4490-4503, doi:10.1093/cercor/bhv076 (2015).

15 Yoris, A. *et al.* in *The Interoceptive Basis of the Mind* (eds Manos Tsakiris & Helena Eds. De Preester) (Oxford University Press, 2018).

16 García-Cordero, I. *et al.* Feeling, learning from, and being aware of inner states: Interoceptive dimensions in neurodegeneration and stroke. *Phil. Trans. R. Soc. B.*, doi:doi: 10.1098/rstb.2016-0006. (2016).

17 Yoris, A. *et al.* The roles of interoceptive sensitivity and metacognitive interoception in panic. *Behavioral and brain functions : BBF* **11**, 14, doi:10.1186/s12993-015-0058-8 (2015).

18 Yoris, A. *et al.* Multilevel convergence of interoceptive impairments in hypertension: New evidence of disrupted body-brain interactions. *Human brain mapping* **39**, 1563-1581, doi:10.1002/hbm.23933 (2018).

19 Couto, B. *et al.* The man who feels two hearts: the different pathways of interoception. *Social cognitive and affective neuroscience* **9**, 1253-1260, doi:10.1093/scan/nst108 (2014).

20 Garfinkel, S. N., Seth, A. K., Barrett, A. B., Suzuki, K. & Critchley, H. D. Knowing your own heart: distinguishing interoceptive accuracy from interoceptive awareness. *Biol Psychol* **104**, 65-74 (2015).

21 Schandry, R. Heart beat perception and emotional experience. *Psychophysiology* **18**, 483-488 (1981).
